# Cellular responses and gene expression profile changes due to bleomycin-induced DNA damage in human fibroblasts in space

**DOI:** 10.1371/journal.pone.0170358

**Published:** 2017-03-01

**Authors:** Tao Lu, Ye Zhang, Yared Kidane, Alan Feiveson, Louis Stodieck, Fathi Karouia, Govindarajan Ramesh, Larry Rohde, Honglu Wu

**Affiliations:** 1 NASA Johnson Space Center, Houston, Texas, United States of America; 2 University of Houston Clear Lake, Houston, Texas, United States of America; 3 NASA Kennedy Space Center, Houston, Texas, United States of America; 4 Wyle Laboratories, Houston, Texas, United States of America; 5 BioServe Space Technologies, Boulder, Colorado, United States of America; 6 NASA Ames Research Center, Moffett Field, California, United States of America; 7 Norfolk State University, Norfolk, Virginia, United States of America; National Taiwan University, TAIWAN

## Abstract

Living organisms in space are constantly exposed to radiation, toxic chemicals or reactive oxygen species generated due to increased levels of environmental and psychological stresses. Understanding the impact of spaceflight factors, microgravity in particular, on cellular responses to DNA damage is essential for assessing the radiation risk for astronauts and the mutation rate in microorganisms. In a study conducted on the International Space Station, confluent human fibroblasts in culture were treated with bleomycin for three hours in the true microgravity environment. The degree of DNA damage was quantified by immunofluorescence staining for γ-H2AX, which is manifested in three types of staining patterns. Although similar percentages of these types of patterns were found between flight and ground cells, there was a slight shift in the distribution of foci counts in the flown cells with countable numbers of γ-H2AX foci. Comparison of the cells in confluent and in exponential growth conditions indicated that the proliferation rate between flight and the ground may be responsible for such a shift. We also performed a microarray analysis of gene expressions in response to bleomycin treatment. A qualitative comparison of the responsive pathways between the flown and ground cells showed similar responses with the p53 network being the top upstream regulator. The microarray data was confirmed with a PCR array analysis containing a set of genes involved in DNA damage signaling; with BBC3, CDKN1A, PCNA and PPM1D being significantly upregulated in both flight and ground cells after bleomycin treatment. Our results suggest that whether microgravity affects DNA damage response in space can be dependent on the cell type and cell growth condition.

## Introduction

Living organisms are exposed to radiation in space that consists of high energy protons and heavy charged particles. For humans, exposure to this environment is expected to cause cancer and other deleterious effects. Current assessment of space radiation risk in astronauts is based on the knowledge gained primarily from human data and animal experiments on the ground under the 1 g gravity condition. If spaceflight factors, microgravity in particular, affect the repair of space radiation-induced damage, then one would expect an additional impact on the mutation rate in living cells [1], and consequently bias current ground-based risk assessment methods. For astronauts, effects of space radiation exposure have been manifested in the increased frequency of chromosome aberrations in the astronauts’ lymphocytes post mission [2–4], early onset of cataracts [5, 6], and light flashes [7, 8].

Potential effects of microgravity on radiation-induced DNA damage repair have been investigated since the early days of human space program. Experiments aimed at addressing such effects were conducted either by exposing cells prior to the launch of samples into space, exposing samples in space, or exposing samples shortly after landing. During an early Germini-3 mission, human lymphocytes in culture were exposed to ^32^P β-particles in space within one hour after reaching the microgravity condition [9]. The cells were kept under the microgravity condition for approximately 4 hours before reentry started. Post-flight analysis of chromosome aberrations in the lymphocytes indicated an increased frequency of β-induced chromosomal deletions in the flight samples in comparison to the ground, whereas the frequencies of dicentrics and rings were similar [9]. However, the potential synergism between microgravity and radiation in chromosomal deletions was not confirmed in a subsequent Germini-XI flight experiment[10]. Inflight exposure of yeast to ^63^Ni β-particles was also performed onboard the Space Shuttle STS-82, with results showing no significant differences in both the induction and the repair of double strand breaks (DSB) between μg and the 1g conditions [11].

In a number of flight experiments, DNA damages were induced by high doses of radiation preflight. The cells were then kept at low temperature after irradiation so that repair of the damages did not occur until they reached orbit. Investigating survival in *S*. *cerevisiae* following this scenario, Pross and colleagues reported possible reduced repair under microgravity in an experiment conducted onboard STS-42 [12], but a repeated experiment conducted onboard STS-76 showed that repair of pre-induced DSB in yeast was not impaired by microgravity [13]. A similar pre-flight exposure experiment using *E*. *coli* and human fibroblasts also found no differences between the flown and ground samples in the kinetics of DNA strand break rejoining [14].

As part of a pre-post flight study, we exposed blood samples collected from an astronaut to γ rays of a set of doses before flight and also within 24 hours after the 9-day STS-103 mission [15]. Comparison of the dose response for total chromosomal exchanges between pre- and post-flight samples did not show an effect of spaceflight. Interestingly, however, Greco and colleagues reported an enhancement of approximately 1.2–2.8 fold in the chromosome aberration frequency in a post-flight astronaut blood sample compared to parallel pre-flight data after exposure to ground based X-rays [16]. Early studies on the combined effects of microgravity and radiation have been reviewed by Horneck [17], and by Pross and Keifer [13], with conclusions that spaceflight does affect the development of organisms, but the majority of spaceflight experiments showed little effects of microgravity on the repair of radiation-induced DNA damage.

In more recent years, experiments have also been conducted using microgravity simulators on the ground, particularly the rotating wall vessels (RWV) [18–20] for cultured cells in suspension, to explore synergisms between microgravity and radiation. In contrast to the flight studies, the majority of ground based experiments demonstrated compromised repair of radiation-induced DNA damages under the simulated microgravity condition. These synergistic effects have been observed in human lymphocytes and human fibroblasts as measured by their survival, and the induction of micronuclei and HPRT mutation [21–22], as well as repair kinetics of γ-H2AX signals [23].However, no effects of simulated microgravity on proton-induced chromosome aberrations in human lymphocytes were observed [24]. Combined effects of radiation and simulated microgravity have also been investigated in rodents using the hindlimb suspension model [25].

To fully address the question of whether the true microgravity condition affects DNA repair with high confidence requires a radiation source in space to induce high levels of DNA damages. Alternatively, high levels of DNA damages can be induced in space by radiomimetic chemicals. In a recent study, we flew human fibroblasts (AG1522) to the International Space Station (ISS) and treated the cells with bleomycin in orbit for 3 hrs. The cells were then fixed for DNA damage analysis and for gene expressions. The cells were then fixed for DNA damage analysis and for quantifying expressions of genes. These cells have been widely used in investigations of radiation damages [26, 27], and were able to be synchronized in mostly the G1 phase upon confluence [28]. In this study, we intentionally induced damages in cells in G1 to minimize the effects of cell growth condition differences between ground and space. Bleomycin is a known chemotherapy drug that induces DNA damages including DSB [29], and has been used previously in spaceflight experiments [30]. The present study is the first to investigate gene expressions in response to DNA damages induced intentionally in space.

## Materials and methods

### Flight hardware

**Fig 1:**
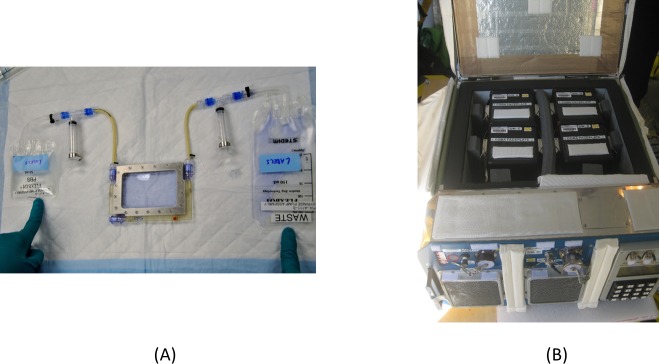
(A) BioCell cell culture chamber (BioServe Space Technologies) used in the flight experiment. Fluid in bags can be transferred in and out of the chamber using syringes. (B) Cells in space were incubated at 37°C in CGBA (BioServe Space Technologies).

BioCells cell culture chambers manufactured by BioServe Space Technologies, University of Colorado, were used to culture the cells in the flight experiments, as shown in Fig 1A. The top surface of the chamber was made of a porous membrane that allowed exchange of O_2_ and CO_2_ in the medium. The cell growing surface was made from the bottom of a T-150 flask (Corning, Corning, NY) that was treated for tissue culture and mounted with seal gaskets. Adherent cells were grown on the bottom surface only. The chamber contains three ports that are connected to bags containing bleomycin, washing, or fixing solutions during the respective steps. Exchange of fluid was achieved with syringes.

Prior to launch, the BioCells were confluent fibroblasts were filled with medium and sealed in a Cell Culture habitat (CHab) that contained 5% CO_2_. The habitat was then placed into a Commercial Generic Bioprocessing Apparatus (CGBA, BioServe Space Technologies, University of Colorado, Boulder, CO) at a pre-set temperature (Fig 1B). Once the cells reached the ISS, the habitat was transferred to another CGBA located onboard the ISS laboratory module.

### Flight cell culture

Normal human foreskin fibroblasts (AG1522, National Institute of Aging) were grown in cultured dishes in a monolayer and the growth is contact-inhibited. Cells of less than 10 passages, routinely cultured at 37^0^ C, 95% humidity, and 5% CO_2_ in α-MEM medium (Invitrogen, Carlsbad, CA) supplemented with 10% fetal bovine serum (FBS) and 50 mg/ml penicillin-streptomycin (Invitrogen, Carlsbad, CA). Cells at passage 6 were frozen and shipped to Kennedy Space Center (KSC) months prior to the scheduled launch date. Two weeks prior to launch, the cells were thawed and plated in T-75 flasks. Seven days prior to the launch date, the cells were seeded in the BioCell cell culture chambers (10 flight and 10 ground control BioCells) at a density of 1x10^6^ cells per BioCell. The cells reached confluence 2 days before launch with about 92% of the cells expected in the G1 phase of the cell cycle. One day prior to the launch, media in the BioCell was replaced with fresh media, and the cell culture chambers were inserted in sealed habitats (5 BioCells per habitat) that were each flushed with CO_2_ (5%) balanced air. The samples were then transported to the launch pad and kept at 20^0^ C. Ground controls were prepared following the exact same procedure used for the flight samples.

### Flight experimental timeline

**Fig 2:**
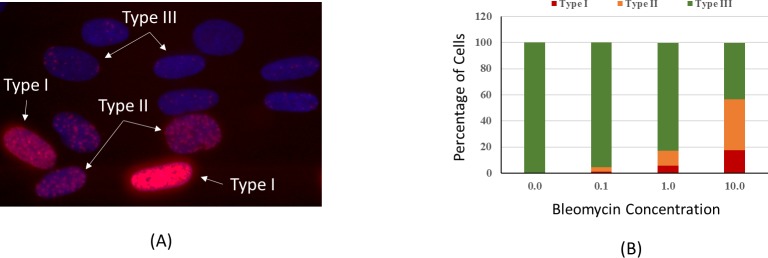
(A) Category of cell nuclei stained with γ-H2AX antibody after bleomycin treatment. Nuclei with pan-nucleated staining are classified as Type I, Nuclei with distinct and countable foci are Type III, and nuclei with staining pattern in between are Type II. (B) Percentage of cells with different types of γ-H2AX staining after bleomycin treatment of 0.1, 1 and 10 mg/ml concentration. The percentage of Types I and II increased as the concentration of bleomycin increased.

On April 18, 2014, our experimental payload was launched from NASA Kennedy Space Center (KSC) in Florida. The cells were kept at 20°C until they reached the ISS. On April 22, the two habitats containing the cells were then transferred to another CGBA preset at 37^0^ C. On April 25, the cells were removed from the CGBA incubator, and the BioCell cell culture chamber was injected with bleomycin at a final concentration of 1 μg/ml or placebo using the same amount of PBS. This concentration is equivalent to exposure of about 0.4 Gy γ-radiation [31], and resulted in a balanced distribution of three types of DNA damages in our ground experiment (Fig 2). The cells were then transferred back to CGBA. After 3 hr incubation with bleomycin, a total of 8 BioCells containing bleomycin or placebo treated cells were removed from the CGBA, washed with PBS, and fixed with RNALater II at a final concentration of approximately 90%. In addition, one BioCell from each treatment group was washed with PBS and fixed with a final concentration of 2% paraformaldehyde (PFA) for 30 min at room temperature before being washed with PBS.

Immediately after fixation, the samples were transferred to a 4°C refrigerator onboard the ISS. On May 18, 2014, the samples were returned to Earth. The samples were kept at 4° C until they arrived at NASA Johnson Space Center (JSC) in Houston, Texas. Total RNA was then isolated using miRNeasy Mini Kit (Qiagen, Valencia, CA) according to the manufacturer’s protocol. The residual genomic DNA was removed using Qiagen RNase free DNase kit.

Processing of the samples on the ISS was performed by an astronaut using a glove bag. The ground control experiment was performed in the same manner, but with a 6-hour offset from the flight schedule at KSC. Cells fixed with paraformaldehyde from spaceflight and ground experiments were rinsed with 1xPBS. Each cell culture plate was separated into 12 pieces and stored in methanol at -20°C.

### Ground experiment with different bleomycin concentrations and with cells in different growth conditions

Confluent AG1522 cells were treated with bleomycin for concentrations of 0.1, 1 and 10 μg/ml for 3 hrs before fixation with 2% PFA for 30 min in order to obtain a dose response relationship, and to determine the concentration used in the flight experiment. To test whether the differences in cellular responses to bleomycin treatment could be attributed to the cell growth condition, AG1522 cells at confluence or in exponentially growth stage were treated with 1 μg/ml bleomycin for 3 hr as in the flight experiment. Cells in these two growth conditions were also fixed with 2% PFA for 30 min. at room temperature at the end of the 3-hr treatment.

### Immunofluorescence analysis

The PFA fixed cells were permeabilized with 0.25% Triton-X-100 in 1x PBS for 15 minutes at room temperature. After blocking with 3% BSA in PBST (1x PBS + 0.5% Tween-20) overnight at 4°C, the samples were incubated with anti- γ-H2AX (1:500, Novus Biologicals, NB100-79967) and appropriate fluorescence-conjugated secondary antibodies. The stained samples, counter stained with DAPI, were mounted with ProLong® Gold Antifade Mountant medium (Life Technologies).

Fluorescence images were captured with a Zeiss Objective EC Plan-Neofluar 40x/1.30 Oil DIC lens on a Zeiss AxioPlan 2 fluorescence microscope in conjunction with Leica CytoVision® software. Images of the same view field were captured at different focal points to ensure accurate counts of foci. Fiji/ImageJ software was used to process images and to count γ-H2AX patterns and foci manually.

Unlike γ-H2AX foci induced by ionizing radiation, bleomycin induces fluorescent image patterns that can be classified into several categories [32]. Similar to the method used after UV irradiation [33], we classified γ-H2AX staining patterns into three categories: pan-nuclear staining (type I), indistinct foci (type II), and distinct countable foci (type III) (Fig 2A). By this definition, untreated cells with a few background foci are classified as Type III. Counting of foci numbers and types were done in three different areas of the BioCell, with a minimum of 10 fields for each sample. In total, over 400 nuclei were counted for each individual treatment condition.

Discrete distributions of cell damage types were compared between flight and ground using a chi-squared contingency table. Distributions of Type III foci counts between samples with differing experimental conditions (ground/space; treated/untreated) were compared non-parametrically with the Mann-Whitney test. Follow-up descriptive summaries of quantiles of the distributions were made with quantile regression [34].

### Microarray analysis

Microarray analysis was performed at the Genomic and NRA Profiling Core facility, Baylor College of Medicine, Houston, Texas. RNA sample quality was verified using a Nanodrop ND-1000 Spectrophotometer (Thermo Scientific) and an Agilent 2100 Bioanalyzer (Agilent), with resulting A260/A280 ratios within a range of 1.95 to 2.03 and RNA Integration Number (RIN) ranging from 9.2 to 10. Then, 50 ng of total RNA was used for RNA amplification and labeling according to the Agilent Quick Amp Labeling Kit (for one-color) Protocol Version 6.7, using Agilent Labeling Kit along with an Agilent’s RNA Spike-In Kit (Agilent). The resulting cRNAs were purified using Qiagen RNeasy mini spin columns and measured again on the Nanodrop for yields (1.1 to 1.5 μg per sample) and dye incorporation (average 13.2 pmol/μg, with 2 of the 15 samples at 7.7 and 9.9 pmol/μg). The samples were then fragmented and 0.6 μg of each hybridization mixture was loaded onto the Human G3 v3 8x60K Expression arrays. The slide was hybridized in Agilent Hybridization Chamber at 65°C with 10 rpm rotation for 17 hours, followed by washing with the Agilent Expression Wash Buffer Set 1 and 2 as per the Agilent protocol. Once dry, the slides were scanned with the Agilent Scanner (G2565BA) using Scanner Version C and Scan Control software version A.8.5.1. Data extraction and quality assessment of the microarray data was completed using Agilent Feature Extraction Software Version 11.0.1.1.

### Microarray data analysis

We applied empirical Bayes methods as described in Linear Models for Microarray Data (LIMMA) [35, 36] to the DNA microarray data to estimate mean expression values, log fold changes and calculate p-values reflecting the differential expression of each gene for the following contrasts: bleomycin treated ground samples versus sham treated ground control (GT_vs_GC), bleomycin treated flight samples versus sham treated flight control (FT_vs_FC), and the interaction (GT–GC) vs (FT–FC). The data were background corrected, and normalized using the normexp and cyclicloess procedures in LIMMA. We filtered out all probe IDs that do not map to known genes. Also using a Perl script that we have developed, we discarded probe IDs that map to non-coding RNA molecules. Then, using the Benjamini-Hochberg procedure to define a p-value threshold such that the false discovery rate (FDR) is controlled to 0.1 or less (q-value < 0.1), differentially expressed genes were chosen for each contrast that met the following selection criteria: q-value < 0.1, average log expression across samples > 6 (which is approximately 1.5 times the background intensity), and absolute log fold change > 0.378 (which is equivalent to a fold change cutoff 1.3). Following this, hierarchical clustering was performed using Pearson correlation metrics and pairwise complete linkage hierarchical clustering algorithm. Gene function and pathway analysis was performed using Ingenuity Pathway Analysis (IPA) software.

### PCR array analysis

Expression levels of a selected number of genes were subjected to verification with the real-time PCR technique. The RT^2^ Profiler PCR array kits designed to analyze 84 genes involved in DNA damage signaling were purchased from Qiagen (Valencia, CA). Total RNAs were reverse-transcribed to cDNAs using RT^2^ First Strand Kit from Qiagen, following manufacturer’s instructions. Real-time PCR reactions were performed on a Bio-Rad CFX96 Real-Time PCR Detection System. Raw data were extracted and analyzed according to Qiagen RT^2^ Profiler Data Analysis Handbook (v3.5). The relative gene expression levels were calculated using the -ΔΔCt method, with normalization performed using expression levels of four house-keeping genes. The list of genes can be found at (http://www.sabiosciences.com/rt_pcr_product/HTML/PAHS-029A.html), and are shown in Table 1. Low abundant RNAs with a cycle number greater than 30 were neglected.

**Table 1:** List of genes involved in DNA damage signaling in the PCR array analysis, and the fold change of expressions after bleomycin treatment in space and on the ground. Fold changes with a PCR cycle number >30 are left blank.

Gene	Ground		Flight		Gene	Ground		Flight	
	Fold	p value	Fold	p value		Fold	p value	Fold	p value
**ABL1**	-1.11	0.21	1.01	0.9	**MPG**	-1.08	0.16	-1.06	0.24
**APEX1**	-1.08	0.34	-1.07	0.15	**MRE11A**	1.04	0.84	-1.01	0.87
**ATM**	1.13	0.68	1.04	0.78	**MSH2**	-1.04	0.9	1.12	0.31
**ATR**	1.04	0.86	1.08	0.6	**MSH3**	1.03	0.83	-1.02	0.84
**ATRIP**					**NBN**	1.05	0.81	-1.05	0.64
**ATRX**	1.24	0.56	-1.11	0.35	**NTH L1**	-1.21	0.03	1.01	0.81
**BARD1**	-1.04	0.83	-1.02	0.78	**OGG1**	1.02	0.65	-1.09	0.23
**BAX**	-1.1	0.18	-1.03	0.45	**PARP1**	1.03	0.77	1.02	0.62
**BBC3**	1.74	0	2.02	0.01	**PCNA**	1.45	0	1.32	0.04
**BLM**					**PMS1**	1.09	0.76	1.02	0.93
**BRCA 1**					**PMS2**	-1.05	0.72	-1.26	0.03
**BRI P1**	1.25	0.52	-1.09	0.61	**PNKP**	-1.22	0.09	-1.01	0.9
**CDC25A**					**PPM1D**	1.98	0	1.77	0
**CDC25C**					**PP P1R15A**	-1.09	0.14	-1.27	0.04
**CDK7**	-1	0.98	-1.08	0.18	**PRKDC**	1.06	0.74	1.04	0.65
**CDKN1A**	1.87	0	2.26	0.07	**RAD1**	-1.06	0.34	-1.07	0.27
**CHEK1**	1	0.97	-1.06	0.5	**RAD17**	1.12	0.61	-1.07	0.55
**CHEK2**	-1.16	0.18	-1.15	0.02	**RAD18**	-1.04	0.77	-1.09	0.15
**CIB1**	-1.12	0.02	-1.02	0.51	**RAD21**	1.01	0.95	-1.04	0.69
**CRY1**	1.06	0.69	-1.07	0.29	**RAD50**	1.06	0.81	-1.05	0.61
**CSNK2A2**	1.07	0.44	1.01	0.82	**RAD51**	-1.06	0.37	1.05	0.61
**DDB1**	-1.04	0.36	1.02	0.66	**RAD51B**	-1	0.94	-1.11	0.02
**DDB2**	1.07	0.25	1.12	0.09	**RA D9A**				
**DDIT3**	1.04	0.64	1	0.95	**RBBP8**	1.03	0.86	1.02	0.88
**ERCC1**	-1.09	0.14	1.02	0.73	**REV1**	1.01	0.97	-1.01	0.94
**ERCC2**	-1.23	0.21	1.03	0.63	**RN F168**	-1.03	0.92	-1.09	0.41
**EXO1**					**RN F8**	1.04	0.74	1.01	0.8
**FA NCA**					**RPA1**	-1.05	0.53	1	0.99
**FANCD2**					**SIRT1**	1.1	0.59	-1.12	0.37
**FANCG**					**SMC1A**	1.13	0.43	1.02	0.8
**FEN1**	-1.07	0.34	1.18	0.2	**SUMO1**	1.08	0.58	-1.07	0.25
**GADD45A**	1.17	0.29	1.17	0.08	**TOPBP 1**	1.12	0.66	-1.05	0.69
**GADD45G**					**TP53**	1.06	0.45	-1.05	0.34
**H2AFX**	-1.07	0.41	1.04	0.72	**TP53BP1**	1	0.99	-1	0.97
**HUS1**	1.05	0.74	-1.1	0.34	**TP73**				
**LI G1**					**UNG**	-1.1	0.32	1.12	0.21
**MAPK12**	-1.18	0.06	-1.05	0.38	**XPA**				
**MBD4**	1.14	0.55	1.05	0.47	**XPC**				
**MCPH1**	1	0.96	-1.02	0.63	**XRCC1**	-1.18	0.07	-1	1
**MDC1**	-1.04	0.82	1.01	0.89	**XRCC2**				
**MLH1**	-1.02	0.95	1.01	0.9	**XRCC3**				
**MLH3**	-1.01	1	-1.13	0.29	**XRCC6**	-1.05	0.42	-1.01	0.77

### Statistical analysis

The distribution of cells with countable foci was analyzed with chi-squared test and Mann -Whitney test for different quantiles.

## Results

### DNA damage quantification

Fig 2B shows the percentages of different staining types for γ-H2AX in AG1522 cells after bleomycin treatment of 0.1, 1.0 and 10.0 μg/ml concentration on the ground. As the bleomycin concentration increased, the percentage of Type I and Type II cells also increased, indicating a correlation between these types of staining and the levels of DNA damage.

**Fig 3:**
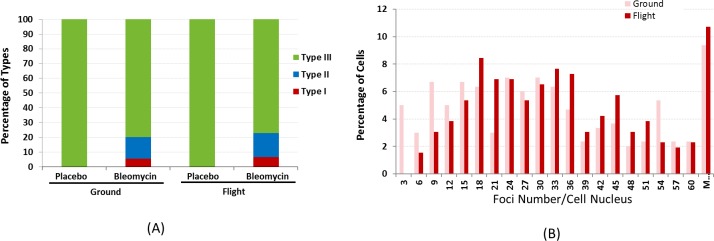
(A) Percentage of nuclei with different types of γ-H2AX staining patterns in the flight and ground samples with and without bleomycin treatment. All of the nuclei were Type III in placebo treated cells, whereas the percentages of Types and II nuclei after bleomycin treatment were similar between the flight and ground samples. For bleomycin treated samples, the percentage of type I, II, and II are 5.5, 14.5, and 80.1 for ground samples, and 6.5, 15.6, and 77.9 for flight samples, respectively. (B) γ-H2AX foci number distribution in Type III nuclei in ground and flight samples after bleomycin treatment showing differences mainly in the lower quantiles of the two count distributions.

The relative contribution of different types of γ-H2AX stained cells after treatment of bleomycin of 1.0 μg/ml for 3 hr are depicted in Fig 3A for both the flight and ground samples. Actual counts and percentages are given in Table 2. Note that all untreated samples exhibited only Type III staining. For bleomycin-treated samples, it can be seen in Fig 3A that the distributions of damage types were very similar for ground and space. Even with the large number of cells in both of these groups (total of 2456, Table 2), there was no evidence of a spaceflight effect on the type of damage (p = 0.58, chi-squared test). As expected, treatment with bleomycin substantially increased the number of Type III foci; from a median count of 3 per cell (untreated, ground) to 28 (treated, ground), and from a median of 3 (untreated, space) to 31 (treated, space). Fig 3B shows the distribution of the number of foci in cell nuclei after bleomycin treatment for both ground and flight. There was evidence that overall, treated cells in space had more damage than their counterparts on the ground (p = 0.020; Mann-Whitney test), with the difference being mainly in the lower quantiles of the two count distributions (Table 3, Fig 3B). For untreated cells, there also appeared to be more damage in space than on the ground, but the difference was not significant (p = 0.125).

**Table 2:** Distribution of damage types (cell counts) by experimental condition (ground (G) or flight (F); treated (T) or untreated(C)).

Type	GC	GT	FC	FT	Total
I	0 (0%)	37 (5.5%)	0 (0%)	43 (6.5%)	80
II	0 (0%)	99 (14.5%)	0 (0%)	104 (15.6%)	203
III	458 (100%)	546 (80.1%)	651 (100%)	518 (77.9%)	2,173
Total	458	682	651	665	2,456

**Table 3:** Estimated 10^th^, 25^th^, 50^th^, 75^th^, and 90^th^ quantiles of Type III foci count distributions for treated cells on the ground and in space. Point estimates 95% confidence limits obtained from quantile regression.

Venue	Percentile	Estimate	95% low	95% upp
Ground	10	7	5.2	8.8
Flight	10	13	11.6	14.4
Ground	25	15	12.2	17.8
Flight	25	20	17.8	22.2
Ground	50	28	25.1	30.9
Flight	50	31	29.3	32.7
Ground	75	45	40.2	49.8
Flight	75	45	41.6	48.4
Ground	90	60	55.2	64.8
Flight	90	62	56.4	67.6

**Fig 4:**
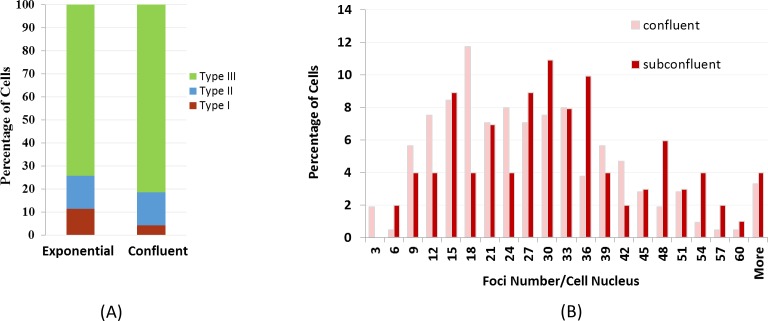
Percentage of nuclei with different types of γ-H2AX staining patterns in exponentially growing and confluent cells after bleomycin treatment of 1 mg/ml on the ground. The percentage of Type I cells was significantly higher in exponentially growing cells. For bleomycin treated samples, the percentage of type I, II, and II are 11.5, 14.2, and 74.3 for exponential samples, and 4.3, 14.4, and 81.3 for confluent samples, respectively. (B) γ-H2AX foci number distribution in Type III nuclei in confluent and exponentially growing cells after bleomycin treatment showing differences in the lower quantiles of the two count distributions.

AG1522 cells in exponentially growing conditions and in confluence were also treated on the ground with 1 μg/ml bleomycin for 3 hrs, and the percentages of different γ-H2AX staining types are shown in Fig 4A. With the same concentration of bleomycin, exponentially growing cells exhibited higher level of Type I staining (11.5%), in comparison to 4.3% Type I staining in confluent cells. However, the Type II stained cells were similar between the two cell growth conditions (14.2% and14.4%). In Type III cells, the distribution of foci number was shifted in the exponentially growing cells, similar to the flight samples in the present study (Fig 4B).

### Microarray analysis

**Fig 5:**
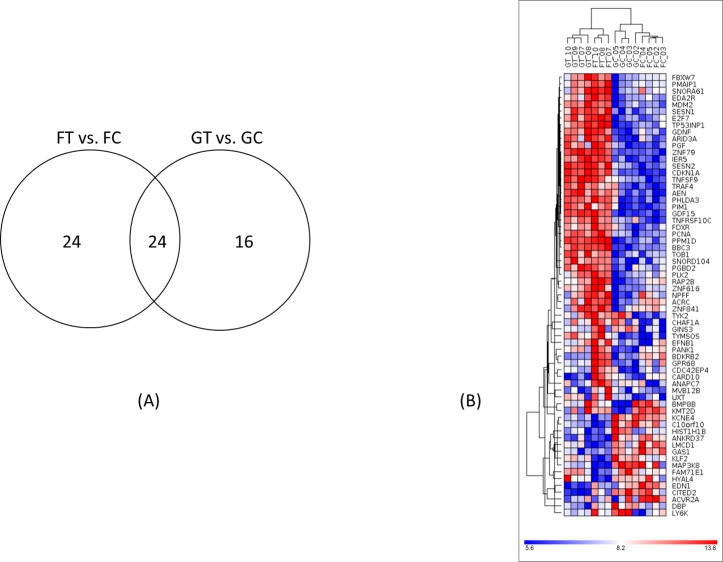
(A) Venn diagram for fibroblasts after bleomycin treatment between flight and ground. (B) Hierarchical clustering of genes that showed significant expression changes after bleomycin treatment in either the ground and flight samples.

For the FT_vs_FC contrast, 48 genes with known functions had expressions significantly altered as a consequence of bleomycin treatment in space. On the other hand, in ground cells treated with bleomycin (GT_vs_GC), 40 genes showed significant expression changes. Among these genes, 24 were common between both contrasts as shown in the Venn diagram (Fig 5A). The heatmap of genes having significant expression changes in either the ground or the flight samples is shown in Fig 5B. Hierarchical clustering of the total of 64 significant genes based on expression values revealed that bleomycin treated samples (irrespective of flight or ground) are grouped together and they are clearly segregated from the control samples. All 64 genes and their fold changes from bleomycin treatment, are listed in Table 4. Despite this clear evidence that genes were responding to the damage caused by bleomycin, there was no indication of any genes responding differently to bleomycin in flight as compared to ground samples (GT–GC) vs. (FT–FC).

**Table 4:** List of common and unique genes whose expressions were significantly changed in flight and on ground after bleomycin treatment.

		Flight			Ground	
	Gene	Fold change	FDR (q-value)	Gene	Fold change	FDR (q-value)
	AEN	1.42	0.018	AEN	1.36	0.036
	ARID3A	1.38	0.031	ARID3A	1.49	0.007
	BBC3	1.84	0.000	BBC3	1.99	0.000
	BTG2	1.98	0.000	BTG2	2.41	0.000
	C10orf10	-1.79	0.042	C10orf10	-1.79	0.034
	CDKN1A	2.17	0.000	CDKN1A	1.96	0.000
	E2F7	1.70	0.033	E2F7	1.60	0.042
	EDA2R	1.46	0.020	EDA2R	1.32	0.079
	FBXW7	1.35	0.046	FBXW7	1.52	0.016
	GDF15	2.71	0.000	GDF15	3.09	0.000
	GDNF	1.33	0.064	GDNF	1.46	0.016
Common	IER5	1.38	0.016	IER5	1.37	0.017
	KCNE4	-1.57	0.042	KCNE4	-1.43	0.079
	MDM2	1.68	0.008	MDM2	1.64	0.016
	PGF	1.72	0.012	PGF	1.45	0.072
	PHLDA3	1.42	0.009	PHLDA3	1.35	0.023
	PMAIP1	1.40	0.009	PMAIP1	1.40	0.017
	PPM1D	1.53	0.000	PPM1D	1.76	0.000
	SESN1	1.55	0.076	SESN1	1.71	0.027
	SESN2	2.24	0.000	SESN2	1.81	0.001
	TNFSF9	1.55	0.008	TNFSF9	1.39	0.024
	TP53INP1	1.61	0.001	TP53INP1	1.75	0.000
	TRAF4	1.34	0.086	TRAF4	1.40	0.045
	ZNF79	1.79	0.000	ZNF79	1.76	0.000
	ACVR2A	-1.32	0.081	ACRC	1.52	0.017
	ANAPC7	1.45	0.034	BMP8B	1.31	0.093
	ANKRD37	-1.34	0.072	CITED2	-1.31	0.021
	BDKRB2	1.35	0.048	DBP	-1.35	0.095
	CARD10	1.36	0.046	SRSF1	1.59	0.024
	CDC42EP4	1.33	0.064	HIST1H1B	-1.56	0.048
	CHAF1A	1.32	0.039	KMT2D	1.35	0.078
	EFNB1	1.48	0.095	LY6K	-1.43	0.094
	FAM71E1	-1.35	0.042	NPFF	1.40	0.027
	FDXR	1.37	0.034	PANK1	1.41	0.016
	GAS1	-1.40	0.041	PGBD2	1.48	0.026
Unique	GINS3	1.35	0.066	PIM1	1.39	0.021
	GPR68	1.31	0.064	PLK2	1.46	0.097
	HYAL4	-1.35	0.010	RAP2B	1.41	0.055
	KLF2	-1.38	0.045	TOB1	1.41	0.024
	LMCD1	-1.31	0.061	ZNF841	1.43	0.048
	MAP3K8	-1.39	0.023			
	MVB12B	1.34	0.046			
	PCNA	1.41	0.004			
	TNFRSF10C	1.39	0.018			
	TYK2	1.39	0.080			
	TYMSOS	1.47	0.018			
	UXT	1.41	0.046			
	ZNF616	1.36	0.022			

**Fig 6:**
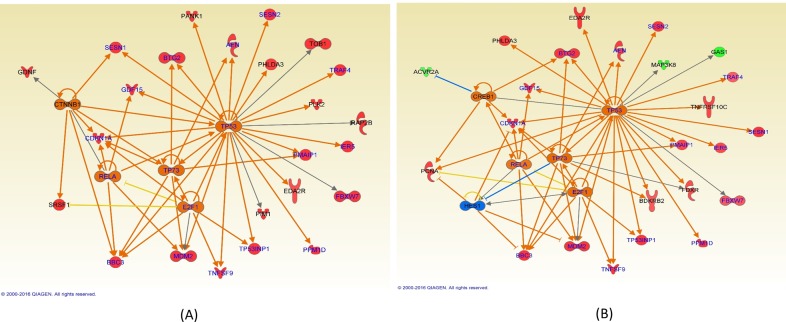
Network in response to the bleomycin treatment in (A) ground and (B) flown cells. TP53, TP73 and RELA are among the common upstream regulators.

Pathway analyses using IPA demonstrated that p53 was the primary pathway responding to the bleomycin treatment in both the flown and ground cells, as shown in Fig 6. In addition, TP73 and RELA were involved in both the flown and ground cells in response to bleomycin treatment. These pathways were among the top 5 significant upstream regulators (Table 5). E2F1 and CDKN2A were also potentially activated as upstream transcription factors. These two upstream regulators have been shown to involve in early responses to ultraviolet radiation-induced DNA damage by directly promoting DNA repair and activating p53 tumor suppressor [37].

**Table 5:** Upstream direct regulators identified by IPA in ground and flown cells in response to the bleomycin treatment.

	Upstream Regulator	Activation z-score	p-value of overlap	Target molecules in dataset
Flight	TP53	4.351	0.0000	AEN,BBC3,BDKRB2,BTG2,CDKN1A,EDA2R,FBXW7,FDXR, GAS1,GDF15,IER5,MAP3K8,MDM2,PCNA,PHLDA3,PMAIP1,PPM1D,SESN1, SESN2,TNFRSF10C,TNFSF9,TP53INP1, TRAF4
	TP73	2.476	0.0000	AEN,BBC3,BDKRB2,BTG2,CDKN1A,FDXR,MDM2,PMAIP1
	CREB1	1.982	0.0238	ACVR2A,BTG2,CDKN1A,PCNA
	RELA	1.932	0.0009	BBC3,BTG2,CDKN1A,GDF15,MDM2
	E2F1	1.907	0.0000	BBC3,CDKN1A,MDM2,PCNA,TNFSF9,TP53INP1,UXT
	HES1	-1.961	0.0000	BBC3,CDKN1A,MDM2,PCNA
Ground	TP53	4.161	0.0000	AEN,BBC3,BTG2,CDKN1A,CITED2,EDA2R,FBXW7,GDF15, IER5,MDM2,PANK1,PHLDA3,PIM1,PLK2,PMAIP1,PPM1D, RAP2B,SESN1,SESN2,TNFSF9, TOB1,TP53INP1,TRAF4
	TP73	2.288	0.0000	AEN,BBC3,BTG2,CDKN1A,MDM2,PMAIP1
	CTNNB1	1.964	0.0059	BBC3,CDKN1A,GDNF,SESN1,SRSF1
	RELA	1.932	0.0004	BBC3,BTG2,CDKN1A,GDF15,MDM2
	E2F1	1.46	0.0000	BBC3,CDKN1A,CITED2,MDM2,PLK2,SRSF1,TNFSF9, TP53INP1

**Fig 7:**
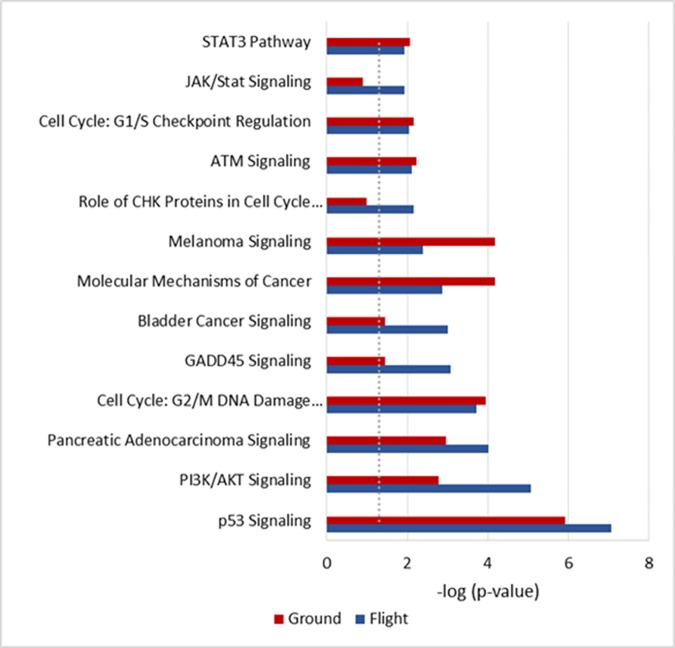
Top Canonical pathways in ground and flown cells in response to the bleomycin treatment.

The IPA analysis also revealed that bleomycin produced similar pathway cascade responses as other genotoxins that induce DNA damage, such as hydrogen peroxide, cisplatin and mitomycin C (Data not shown). Canonical pathway analysis indicated that other DNA damage signaling pathways, such as ATM and the cell cycle checkpoint, also play a role under both gravity conditions (Fig 7). Although several pathways, such as CREB1, CTNNB1, and HES1, were recognized when the unique genes identified in either flight or ground (Table 4) were input into IPA, these pathways failed to reach any statistical significance.

### PCR array analysis of DNA damage signaling genes

**Fig 8:**
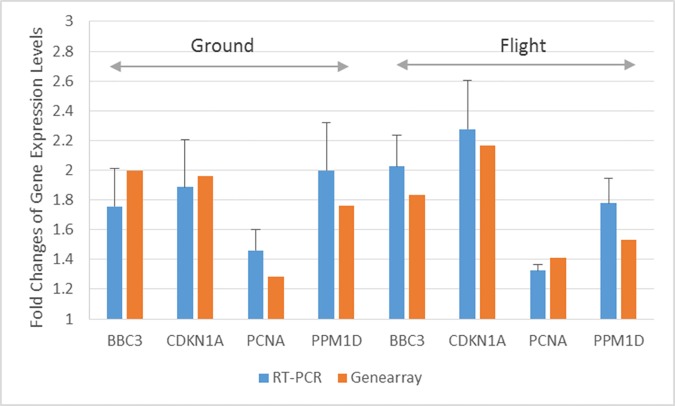
Comparison of fold changes of expressions of genes in Table 1 between microarray and PCR analysis in the flown and ground cells after bleomycin treatment. Only significant changes (Fold > 1.3 and FDR<0.1 for microarray, and Fold >1.3 and P<0.05 for PCR array) are listed. BBC3, CDKN1A, PCNA and PPMID were the only genes up-regulated in all four cases.

To validate the gene microarray data, PCR arrays were performed for a set of genes specifically involved in DNA damage signaling pathways. Of the 84 genes, BBC3, CDKN1A, PCNA and PPM1D were the only four genes having significant expression changes in both flight and ground samples after bleomycin treatment. The fold changes of expression values and p values of these DNA damage signaling genes are listed in Table 1. Comparison of the genes having significant expression changes in the microarray analysis is shown in Fig 8, indicating an agreement between the microarray and PCR array data.

## Discussion

Whether spaceflight factors, microgravity in particular, affect cellular responses to DNA damages is a critical question to be addressed for human space exploration. In this flight study, we induced DNA damage intentionally on the ISS with bleomycin and investigated early responses by measuring the phosphorylation of histone protein H2AX for quantification of DNA damages, and by analyzing gene expressions using both the microarray and the PCR array methods. By classifying γ-H2AX staining patterns in different types, we found a clear increase in the percentage of cell nuclei with pan-nucleus staining (Type I) as the concentration of bleomycin increased (Fig 2). Comparison of different types of γ-H2AX staining patterns in cells after bleomycin treatment between flight and ground revealed a similar percentages of Type I (~6%) and Type II (~15%) cells, and the percentages were not significantly different (Tables 2). However, detailed counting of the number of foci in the countable γ-H2AX stained cells (Type III) indicated differences in the low quantiles of the distribution in cells after bleomycin treatment (Table 3, Fig 2B). In the present study, the cells were fixed at one time point of 3 hours after bleomycin treatment, due primarily to the limitation of samples allowed in a spaceflight experiment. This is typically the time point optimal for investigations of gene expressions. With acute damage to cells by ionizing radiation, it is known that the γ-H2AX intensity would peak within one hour post irradiation, and decrease as the DNA damage is repaired [38]. In the present study, the cells were treated continuously with bleomycin, and the γ-H2AX signals reflected a mix of accumulated damage over the 3 hr period and the repair of some of the damages [32]. Unlike ionizing radiation, γ-H2AX foci induced by toxic chemicals or UV may not necessarily represent DNA double strand breaks [33]. These cells in G1 phase of the cell cycle do not go through apoptosis.

In a previous publication, we reported that AG1522 cells in space proliferated slightly faster [28]. To determine whether cell growth condition was responsible for the γ-H2AX foci count in Type I nuclei between flight and ground, we treated exponentially growing and confluent cells, on the ground, with bleomycin. We showed that levels of bleomycin-induced DNA damages varied with the cell growth condition in that the percentage of Type I γ-H2AX stained cells were significantly higher in exponentially growing cells (Fig 4A). Differences in the foci number distribution was also observed between the cells in these two different growth conditions. Although cells in space in our flight experiment were far from exponential growing conditions, cell growth may be partly responsible for the different foci distributions between flight and ground as shown in Fig 3B. It has been reported that human colon carcinoma cells treated with 0.5 μg/ml bleomycin for 2 days in space on board Space Shuttle STS-95 showed a 4-fold increased mutation rate in comparison to the ground controls [30], but it was not clear whether the different response in space was due to the cell growth condition. It should also be noted that in the colon carcinoma study, the cells were incubated with bleomycin even during the reentry before the samples were harvested on the ground [30]. In our study, faster proliferation in space may be the result of microgravity, or hypergravity and vibration during launch, or a combination of different factors.

In the present study, we also investigated gene expression profiles using the microarray technique in cells after bleomycin treatment with the intention of identifying distinct responses at the molecular level to DNA damage in space. Hierarchical clustering of genes with significant expression changes (Table 4, Fig 5) revealed that bleomycin treated samples for both the flight and ground are grouped together and they are clearly segregated from the control samples. In both the ground and flown cells, activation of the p53 pathway was the top response (Fig 6, Table 5). p53, as well as its downstream regulators such as CDKN1A, is known to be induced in cultured cells or in animals after DNA damage [39–41]. Cancer cells deficient in the p53 function are also known to have altered cell killing effects of bleomycin [42, 43]. In addition to p53 signaling, other signaling pathways that are known to respond to DNA damages [44], including ATM, G1/S and G2/M cell cycle checkpoint signaling, were also found to be common in the flown and ground cells in response to the bleomycin treatment (Fig 7). The similar effects of bleomycin for both flight and ground samples were confirmed quantitatively in that there were no genes with significant differences with respect to the interaction contrast (GT–GC) vs (FT–FC).

We also analyze the expressions of genes involved in DNA damage response using PCR arrays, and found that only BBC3, CDKN1A, PCNA and PPM1D were upregulated after bleomycin treatment in either the ground or flown samples treatment; a finding which agreed well with the microarray data analysis results (Fig 7). The expressions of BCL2 Binding Component 3 (BBC3), Cyclin Dependent Kinase Inhibitor 1A (CDKN1A, p21), Proliferating Cell Nuclear Antigen (PCNA), and Protein Phosphatase, Mg2+/Mn2+ Dependent 1D (PPM1D) are all induced in a p53-dependent manner in response to various environmental stresses. BBC3 encoded protein is a member of the BCL-2 family, and is an essential mediator for p53-dependent and p53-independent apoptosis [45]. PCNA is expressed in the nucleus, and is involved in a number of DNA repair pathways including the RAD6-dependent DNA repair pathway and non-homologous end-joining repair pathway [46]. Cells treated with bleomycin have been reported to display PCNA foci [47]. PPM1D is a member of the PP2C family of serine/threonine protein phosphatases. This phosphatase reduces the phosphorylation of p53, negatively regulates the activity of p38 MAP kinase, and suppresses p53-mediated transcription and apoptosis [48]. It is activated in human cells in response to damage from ionizing radiation and UV [49]. CDKN1A is a potent cyclin-dependent kinase inhibitor and cell cycle regulator [50]. Its encoded protein mediates the p53-dependent cell cycle G1 phase arrest in response to a variety of DNA damages [51]. This protein can interact with PCNA to play a regulatory role in S phase DNA replication and DNA damage repair [52]. Although H2AX was phosphorylated in both the flight and ground samples after bleomycin treatment, no significant increase of the RNA level of the H2A histone family X gene (H2AFX) was observed. Together with the microarray data, our results suggest no differential responses at the molecular level to bleomycin-induced DNA damages in confluent human fibroblasts between flight and ground.

Previously, we reported that in AG1522 cells flown to the ISS without treatment of toxic chemicals, cells in space showed activation of NF-κB which triggered activation of a number of growth factors such as HGF and VEGF [28]. Here, we also compared the gene expressions in the flight samples in respect to the ground controls, either treated or untreated with bleomycin. Using the same fold change and FDR thresholds (Fold > 1.3 and FDR <0.1), a total number of 697 and 432 genes had significant expression changes in space in the untreated (FC-GC) and treated samples (FT-GT), respectively. Analysis of these genes using IPA confirms that NF-κB was the top canonical pathways in both the treated and untreated groups (Table 6). It should be noted that in Zhang et al. [28], cells were removed from the incubator and fixed immediately, whereas in the present study, the cells were removed from incubator, treated with placebo or bleomycin, transferred back to incubator for 3 hrs, and fixed afterwards.

**Table 6:** Top three canonical pathways sorted by the z-score in cells flown in space in comparison to the ground with or without treatment of bleomycin.

Canonical Pathway	FC vs. GC	FT vs. GT
NF-κB Signaling	2.33	2.00
Intrinsic Prothrombin Activation Pathway	2.00	2.00
Wnt/Ca+ pathway	2.00	2.00

The RNA levels in the PCR array analysis were also compared between the flight and ground samples for determination of changes of gene expressions due to the spaceflight condition alone. Of the 84 genes, none had significant expression changes in space in comparison to the ground, either in bleomycin or placebo treated samples, indicating that spaceflight condition did not alter the baseline level of these RNAs involved in DNA damage signaling. In human fibroblasts, spaceflight alone had affected the pathways mostly involved in cell proliferation [28].

### Conclusions

Experiments conducted in space that were aimed at addressing the question of the effects of spaceflight on the repair of DNA damages induced by X- rays, γrays, or β particles have so far produced conflicting results, with more flight studies showing no synergism between microgravity and radiation exposure [13, 17]. Our present results suggested that for human fibroblasts in mostly G1 phase of the cell cycle, early responses to bleomycin-induced DNA damage, as measured by the percentages of types of immunofluorescence staining of γ-H2AX, were similar between space and ground. The difference in the foci number distribution may be due to the faster proliferation of cells in space. Future capability in microscopy to detect cell cycle progression in space will allow confirmation of this conclusion. The primary response to bleomycin treatment was in the p53 pathway in both gravity conditions, as indicated from the microarray analysis and confirmed with the expression of genes involved in DNA damage signaling. Other DNA damage-signaling pathways, such as ATM and cell cycle checkpoint, were also similar between flight and ground. Our results suggest that if all of the cells were in G0/G1 phase of the cell cycle under the same growth condition, microgravity would pose minimal effects in cellular and molecular responses to DNA damages. Such results would have significant impacts on accessing the health risks associated with spaceflight. Cells in living organisms in the true microgravity environment still experience mechanical loading from varying blood pressures that cells in a culture dish do not experience, and could respond differently to DNA damage.
